# Author Correction: On the thermodynamic origin of metabolic scaling

**DOI:** 10.1038/s41598-018-30973-x

**Published:** 2018-08-21

**Authors:** Fernando J. Ballesteros, Vicent J. Martinez, Bartolo Luque, Lucas Lacasa, Enric Valor, Andrés Moya

**Affiliations:** 1 Observatori Astronòmic, Universitat de València, Parque Científico de la Universitat de València, Paterna, Spain; 2 Departamento de Matemática Aplicada y Estadística, ETSI Aeronauticos, Universidad Politécnica de Madrid, Madrid, Spain; 3 School of Mathematical Sciences, Queen Mary University of London, Mile End Road, London, E14NS UK; 4 Departament de Física de la Terra i Termodinàmica, Universitat de València, Valencia, Spain; 5 Instituto de Biología Integrativa de Sistemas, Universitat de València-CSIC, Parque Científico de la Universitat de València, Paterna, Spain

Correction to: *Scientific Reports* 10.1038/s41598-018-19853-6, published online 23 January 2018

This Article contains an error in the Author Contributions section.

“This work has been funded by projects AYA2013-48623-C2-2, FIS2013-41057-P, CGL2013-46862-C2-1-P and SAF2015-65878-R from the Spanish Ministerio de Economa y Competitividad and PrometeoII/2014/086, PrometeoII/2014/060 and PrometeoII/2014/065 from the Generalitat Valenciana (Spain).”

should read:

“This work has been funded by projects AYA2016-81065-C2-2, FIS2013-41057-P, CGL2013-46862-C2-1-P and SAF2015-65878-R from the Spanish Ministerio de Economa y Competitividad and PrometeoII/2014/086, PrometeoII/2014/060 and PrometeoII/2014/065 from the Generalitat Valenciana (Spain).”

